# Navigating Diagnosis and Treatment Dilemmas in Visceral Leishmaniasis Reactivation in a HIV-Positive Patient

**DOI:** 10.7759/cureus.68902

**Published:** 2024-09-07

**Authors:** Dinusha Gayathri, Buddhika Dhananjalee Alahakoon, Udaya Ralapanawa, Ganga Pathirana, Godayalage Chamari Madushani Weerasooriya

**Affiliations:** 1 General Medicine, Postgraduate Institute of Medicine, Colombo, LKA; 2 General Medicine, Teaching Hospital Peradeniya, Peradeniya, LKA; 3 Faculty of Medicine, University of Peradeniya, Peradeniya, LKA; 4 Venereologist, Teaching Hospital Kandy, Kandy, LKA; 5 Hematology, Postgraduate Institute of Medicine, Colombo, LKA

**Keywords:** dilemmas, hiv positive, reactivation, treatment, visceral leishmaniasis

## Abstract

Reactivation of visceral leishmaniasis (VL) in human immunodeficiency virus (HIV)-positive patients poses complex management challenges, requiring tailored treatment approaches and robust follow-up strategies. There are very few case reports of visceral leishmaniasis (VL) published in Sri Lanka as of today.

Here, we present a case of a 38-year-old, HIV-positive male who was treated for a reactivation of VL. He presented with a prolonged febrile illness without an identifiable infection focus. The evaluation confirmed VL reactivation. Using current evidence-based guidelines, our patient was treated with intravenous liposomal amphotericin B and achieved a parasitological cure.

By exploring the challenges associated with managing VL among HIV-positive individuals, we emphasize the importance of reliable follow-up protocols and investigations to assess treatment success, ensuring optimal patient outcomes.

## Introduction

Leishmaniasis is one of the neglected tropical diseases that affects millions of people worldwide. It is caused by the Leishmania parasite and transmitted by female sandflies.

Human immunodeficiency virus (HIV)-associated visceral leishmaniasis (VL/HIV) is becoming a major problem, especially in East Africa, Brazil, and India [1]. In contrast to VL/HIV-infected patients without human immunodeficiency virus (HIV)/acquired immune deficiency syndrome (AIDS), VL/HIV-co-infected patients exhibit a less robust clinical response to treatment and more relapses, death, and drug toxicity [1].

Despite high relapse rates and difficulties eradicating leishmania from HIV-infected patients, only limited evidence exists regarding the best treatment strategies after relapse [2,3]. The available evidence suggests that amphotericin B is superior to other anti-leishmaniasis treatments for HIV-infected patients with visceral leishmaniasis [3]. There is still a great deal of uncertainty regarding amphotericin's optimal dose and the difference in efficacy between its various formulations.

For individuals with VL/HIV coinfection in Southeast Asia, the World Health Organization (WHO) panel recommends liposomal amphotericin B (L-AMB) + miltefosine instead of L-AMB monotherapy [4]. However, further clinical study of the use of combination therapy in VL/HIV coinfection remains a necessity due to the uncertainty of the currently available evidence.

In spite of higher relapse rates than in HIV-negative people, more research is needed to clarify questions such as the role of combination therapy, relapse prevention, and post-treatment monitoring.

## Case presentation

Our patient is a 38-year-old male, previously diagnosed with visceral leishmaniasis and retroviral infection at the age of 36 while investigating for prolonged fever, presented with a history of low-grade fever that didn’t respond to over-the-counter paracetamol. He was also complaining of generalized malaise and loss of appetite for two weeks. However, he did not experience weight loss or a history suggestive of tuberculosis. Other than that, his systemic review was unremarkable. There was frequent travel history to North Central province, Polonnaruwa, his hometown, but there was no travel history out of the country. Other than highly active antiretroviral therapy (HAART) for the retroviral infection, his drug history was unremarkable. Splenomegaly was the only notable finding on the examination. His admission investigations are listed in Table 1.

**Table 1:** Investigations of the patient. CRP: C-reactive protein, ESR: erythrocyte sedimentation rate.

Investigation	Results
Total white cell count	5.18×10^9^/L
Neutrophils	2.59×10^9^/L
Lymphocytes	1.83×10^9^/L
Eosinophils	0.21×10^9^/L
Basophils	0.01×10^9^/L
Monocytes	0.54×10^9^/L
Hemoglobin	10 g/dl
Platelets	173,000 u/L
CRP	19.7 mg/dl
ESR	15 mm/h

**Figure 1:**
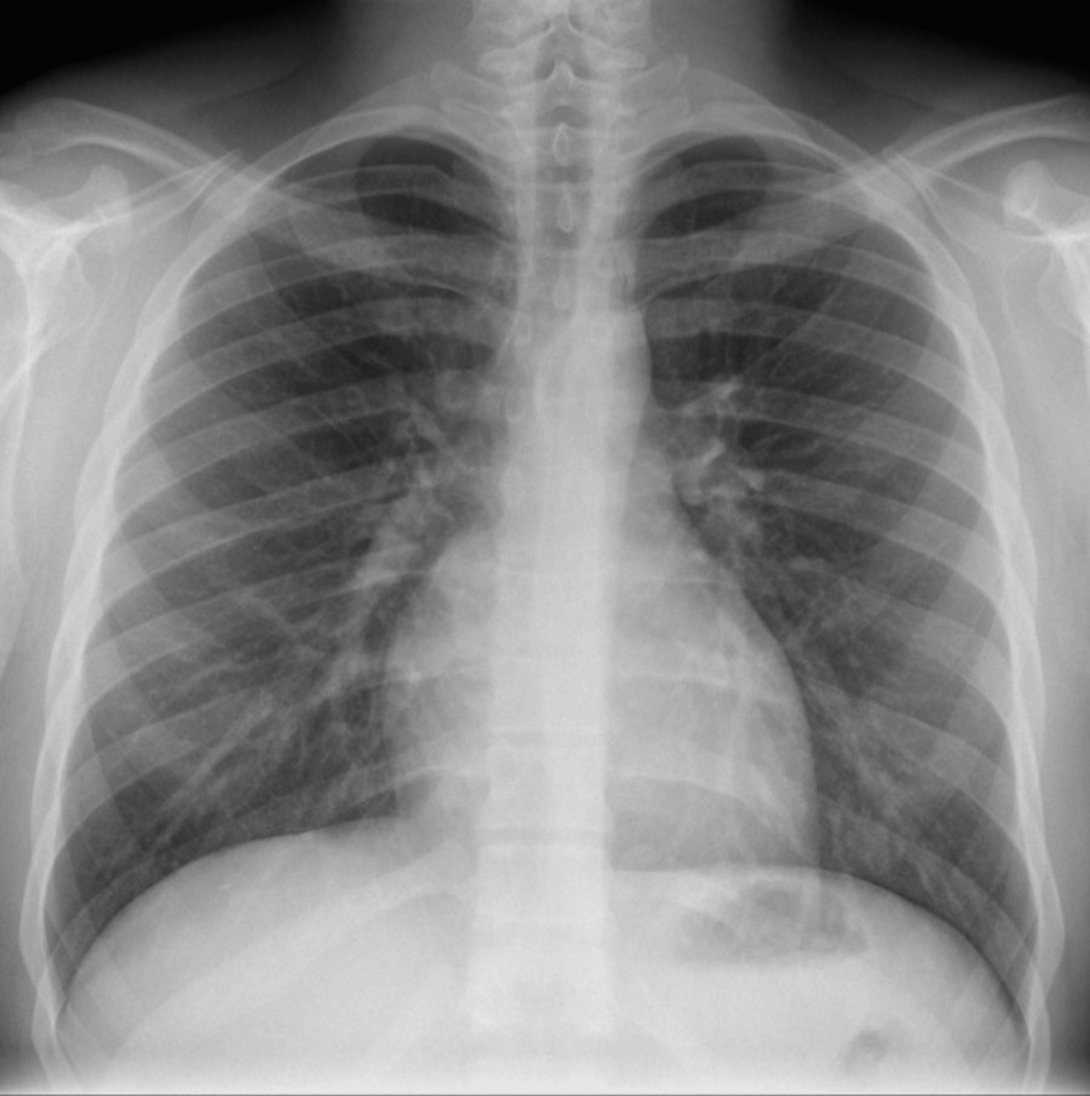
Chest X-ray of the patient.

There were normochromic normocytic cells and hypochromic microcytic cells in the blood picture, as well as white blood cells; platelet morphologies were normal. Urine full report, blood culture, and urine culture were normal. Chest X-ray revealed no abnormalities (Figure 1).

In the ultrasound scan of the abdomen, there was moderate splenomegaly but no hepatomegaly or intraabdominal lymph nodes. The 2D echocardiogram revealed no vegetation, and all heart valves were normal.

However, it was noted that regardless of proper adherence to HAART, his cluster of differentiation 4 (CD4) count has always been low, with an absolute count of 179 at this presentation. It was also noted that the treating venereologist had suggested starting secondary prophylaxis after the first diagnosis of visceral leishmaniasis in the background of a low CD4 count. 

**Figure 2:**
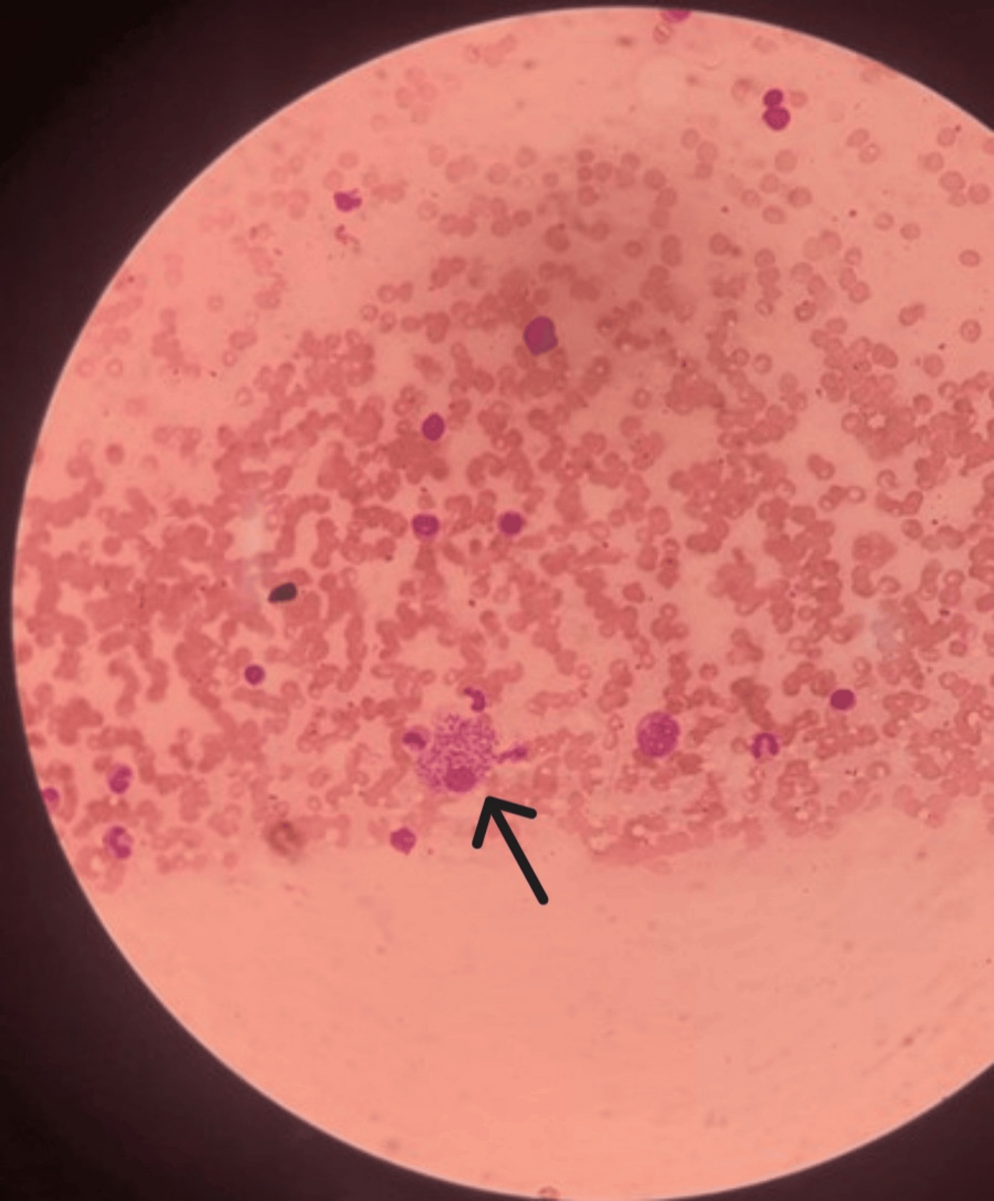
Black arrow showing Leishmania donovani bodies in bone marrow sample.

In the presence of underlying immune deficiency, fever, and loss of appetite, Mantoux and sputum acid-fast bacillus (AFB) were performed, which resulted in negative. In the presence of fever, splenomegaly, and the history of leishmaniasis, the possibility of reactivation of leishmaniasis was entertained. Therefore, bone marrow was performed, which showed inclusions in macrophages suggestive of *Leishmania donovani* bodies (Figure 2). Thus, the diagnosis of reactivation of leishmaniasis was made.

The dilemma we faced was the finding of the best treatment protocol for visceral leishmaniasis reactivation in an HIV-positive patient. According to available data, we found that consensus has not been reached regarding the optimal therapy for VL [5]. We discussed with the experts in the field to find the best treatment strategy for our patients. Evidence supported the use of L-AMB as the primary therapy for patients with VL/HIV [6,7].

The next challenge was to determine the optimal dose and duration of the therapy. His initial episode was treated with intravenous (IV) L-AMB 200 mg daily for five days (2-4 mg/kg/day based on body weight of 53 kg), followed by 200 mg on days 10, 17, 24, 31, and 38. Considering the reactivation following this regime, it was decided by a multidisciplinary team comprising a parasitologist, microbiologist, and clinical pharmacologist to treat him with IV L-AMB 250 mg daily (5 mg/kg/day based on body weight of 50 kg) for five days followed by 250 mg weekly for five weeks. 

Our patient exhibited a drastic clinical improvement with L-AMB. Two weeks after the completion of treatment, a repeat bone marrow examination was performed, and it was free of Donovan bodies, confirming a parasitological cure. However, his CD4 count persistently remained persistently low, below 350.

## Discussion

Leishmaniasis is a vector-born parasitic infection with three main clinical entities. They are cutaneous-the most common form, visceral-the most severe form, and mucocutaneous-the most destructive form. The disease is endemic in over 94 countries in Africa, Asia, the Americas, and Europe [8].

Visceral leishmaniasis (VL) is caused by *L. donovani* and *L. infantum* parasites. *L. donovani *is endemic in Southeast Asia and East Africa. *L. infantum *is endemic in Southern Europe, North Africa, West Central Asia, and the Americas. Locally acquired visceral leishmaniasis was first reported in Sri Lanka in 2007 [9], ​​​and Sri Lanka declared leishmaniasis a notifiable disease in 2008.

Notably, HIV infection, immune suppression, and malnutrition predispose to the disease. HIV-associated visceral leishmaniasis (VL/HIV) is becoming a significant problem in East Africa, Brazil, and India.

Typical features of VL include prolonged fever, hepatosplenomegaly, weight loss, and pancytopenia. HIV and VL both affect T-cell-mediated immunity and reduce the effectiveness of immune responses in VL/HIV patients in a synergistic manner. Thus, VL/HIV patients can present with atypical symptoms. Thus, clinicians should be extremely suspicious of atypical VL when assessing HIV-positive, symptomatic individuals from areas where leishmaniasis is endemic.

Due to impaired immune mechanisms, VL/HIV patients show a higher frequency of relapses [1]. With multiple relapses, fever can be a less prominent feature, and most patients present with isolated splenomegaly, leading to delayed diagnosis and poor outcome [2]. Currently, no vaccine is available to prevent leishmania infection. Hence, disease control is challenging due to limiting access to diagnostic and treatment services.

The gold-stranded diagnostic test for VL is the detection of parasites in splenic biopsies or bone marrow. Even though they are painful and invasive, they are capable of diagnosis and evaluate the response to treatment reliably.

Detection of leishmania antigens or antileishmanial antibodies in blood samples is used to make an immunological diagnosis. Despite being treated, leishmania-specific antibodies persist in the blood of infected individuals for years. Therefore, serum-based antibody detection tests cannot be used to determine a cure [10,11]. Several studies have been conducted to overcome these limitations, using saliva or urine samples instead of serum for VL diagnosis. One Indian study showed promising results of using urine-based dipstick assay and enzyme-linked immunoassay (ELISA) for diagnosis of VL [1]. They suggest a urine dipstick test to assess the cure. However, the diagnostic value of urine-based ELISA and dipstick tests warrants further longitudinal studies. In endemic areas, the rk39 ELISA and direct agglutination test (DAT) have been extensively validated to document VL as immunological tests [12].

Different molecular detection methods have been developed to target specific DNA and/or RNA genes. Polymerase chain reaction (PCR) and real-time quantitative PCR (qPCR) are the most rewarding techniques found so far.

Most VL/HIV patients do not exhibit detectable antibodies, making serological tests ineffective. HIV coinfection is associated with higher levels of parasitemia, so direct detection by PCR or qPCR of parasites or their components is increasingly used not only for diagnosis but also for treatment monitoring. The availability of these tests is often limited in poor healthcare settings. As of now, there is no clear evidence to support recommendations on VL/HIV serological or molecular diagnosis [12].

Hence, the detection of parasites in bone marrow is the only reliable test for diagnosis and follow-up for VL/HIV patients so far. Therefore, a non-invasive test with acceptable sensitivity and competency to diagnose and follow up would be an asset for VL/HIV patients.

The treatment of leishmania can become complicated due to various drug resistance. Due to its high biological heterogeneity, we have not found a universally effective treatment option for this disease. The most effective chemotherapeutic agents against primary and secondary VL are amphotericin B, pentavalent antimonials, paromomycin (a parenteral aminoglycoside), and miltefosine (the first oral drug to treat VL). Initially, antimonial drugs were recommended by WHO as first-line drugs but are gradually being replaced by L-AMB as a result of multiple toxicities and ineffectiveness associated with resistance [3,5].

The available evidence suggests that amphotericin B is superior to other treatments for HIV-infected patients with visceral leishmaniasis VL/HIV [3]. As stated by WHO, the standard therapy for VL/HIV is IV amphoteric B. WHO's recommended dose regime is IV L-AMB 3-5 mg/kg daily, 10 doses or intermittently on days one to five and on days 10, 17, 24, 31, and 38 [8]. We used this treatment regime to treat our patient, and he responded well.

A majority of the evidence for L-AMB comes from Mediterranean countries where *L. infantum *is causing the disease. *L. infantum *in Mediterranean countries exhibits different virulence and drug susceptibility than African and Asian *L. donovani*. Only a few studies have been conducted to assess the efficacy of current treatments in Asia and Africa. India is the only source of data from Asia at the moment [6]. Therefore, optimal treatment regimens have not yet been determined, and further studies are needed.

Recent studies suggested that a combination of L-AMB and miltefosine may be more effective than monotherapy in VL/HIV patients due to the increased drug resistance observed with monotherapy [3,7]. WHO suggests miltefosine (100 mg/day for 14 days) together with L-AMB (up to 30 mg/kg, given at a dose of 5 mg/kg on days one, three, five, seven, nine, and 11) as a combination therapy [4]. However, further clinical trials of combination therapy in VL-HIV coinfection remain a necessity due to the very low certainty of the currently available evidence.

In HIV infection and VL, chronic cellular activation is a crucial immunopathogenic mechanism. Thus, VL/HIV patients have elevated levels of activated CD38+ and CD8+ T lymphocytes and pro-inflammatory cytokines. This enhanced cellular activity remains despite successful control of the viral and parasite load. Thus, making VL/HIV patients more susceptible to recurrent relapses. In this context, evidence suggests that secondary prophylaxis for leishmaniasis helps to maintain remission of VL in HIV patients [1].

In Southeast Asian patients with VL/HIV, the WHO panel recommends secondary prophylaxis after the first episode of VL. Liposomal amphotericin B at 3-5 mg/kg per day every three to four weeks or amphotericin B deoxycholate at 1 mg/kg every three to four weeks are the recommended regimes for this purpose [4]. If CD4 cells remain above 350 cells/mm^3^ for at least six months, or if the HIV viral load is undetectable and there is no relapse of VL, prophylaxis can be stopped. Due to limited evidence, further studies are needed to establish a universal criterion.

Even though our patient exhibited both a clinical and parasitological cure, his CD4 values remained low. In the absence of proper consensus, a combined decision was taken to closely monitor him during follow-up without starting prophylaxis.

## Conclusions

Managing leishmaniasis in HIV-positive patients presents significant challenges due to their suboptimal response to treatment, susceptibility to multiple relapses, and emergence of drug resistance. Current literature highlights several outstanding questions regarding the optimal dosage of medication, the role of combination therapy, and relapse prevention strategies. A lack of available data underscores the need for further research to address these gaps and provide evidence-based guidelines for VL/HIV co-infection management. The establishment of national programs aimed at identifying relapses and implementing post-treatment monitoring protocols is imperative to improve patient outcomes and reduce disease burden.
